# Effect of aging and lifestyle on photoreceptors and retinal pigment epithelium: cross-sectional study in a healthy Danish population

**DOI:** 10.1080/20010001.2017.1398016

**Published:** 2017-11-05

**Authors:** Jacob Harris, Yousif Subhi, Torben L. Sørensen

**Affiliations:** ^a^ Clinical Eye Research Division, Department of Ophthalmology, Zealand University Hospital, Roskilde, Denmark; ^b^ Faculty of Health and Medical Sciences, University of Copenhagen, Copenhagen, Denmark

**Keywords:** Aging, lifestyle, photoreceptors, retinal pigment epithelium, optical coherence tomography

## Abstract

Photoreceptors and their supporting retinal pigment epithelium constitute the key functional parts of the retina. Here, a study was undertaken to show how aging and lifestyle factors affect the photoreceptor layer and the retinal pigment epithelium and Bruch’s membrane complex (RPE-BM) *in vivo* in a healthy Danish population using spectral-domain optical coherence tomography. This was a cross-sectional study of healthy humans aged ≥50 years. All participants were interviewed for medical history and lifestyle factors. Maculae of all participants were scanned using spectral-domain optical coherence tomography. The thickness of the photoreceptor layer and the RPE-BM was measured on one eye from each participant. In 150 eyes of 150 participants, it was found that aging was associated with a decrease in the thickness of the photoreceptor layer (−0.143 μm/year, P = 0.031) and an increase in the thickness of the RPE-BM layer (0.100 μm/year, P = 0.029) at the foveal minimum. Regarding lifestyle factors, alcohol intake or BMI were not associated with any significant trend, but physical inactivity and smoking had effects on the photoreceptor layer (decreased thickness) and the RPE-BM layer (increased thickness) indicating an accelerated aging process of the macula. Taken together, aging affects photoreceptors and the RPE-BM, and these aging trends are accelerated in smokers and the physically inactive.

## Introduction

Photoreceptors and the retinal pigment epithelium (RPE) constitute the starting point of the visual system. Photoreceptors react upon light stimulus whereas the RPE supports the tightly packed and metabolic-demanding photoreceptors allowing a high-resolution vision. Photoreceptors are elongated cone- or rod-shaped cells that synapse with bipolar cells of the inner retina to pass on information on light stimulus. The RPE consists of a monolayer of highly pigmented cubical cells. Apically, RPE microvilli extend and interact with the photoreceptor tips for support. Basally, RPE is in close contact with Bruch’s membrane for barrier function forming an RPE-Bruch’s membrane (RPE-BM) complex. Aging and dysfunction of this system is closely linked to diseases such as age-related macular degeneration, which is the leading cause of irreversible vision loss in the developed world [1,2]. Risk factors include lifestyle factors such as physical inactivity and smoking [2,3].

Aging of the photoreceptors and the RPE-BM are well-characterized from physiological studies, studies *in vitro*, and studies on mice [4,5]. We investigated photoreceptors and RPE-BM in elderly humans *in vivo* using optical coherence tomography (OCT). In this study, we evaluated how aging and lifestyle factors correlate with thicknesses of the photoreceptor layer and the RPE-BM layer in a healthy Danish population consisting of 150 aged participants.

## Methods

This was a single-center cross-sectional study of elderly healthy participants aged ≥50 years. Oral and written informed consent was obtained prior to participation. The study was approved by the Regional Committee of Ethics in Research of the Region of Zealand (Journal No. SJ-142, SJ-379, and SJ-385) and adhered to the tenets of the Declaration of Helsinki. Blood samples from some of the participants were used for studies of aging and age-related macular degeneration [6–9].

### Participant eligibility

Participants were recruited from healthy visitors to our department. All participants were subject to an interview on medical history. Medical history was crosschecked with the electronic patient journal. Participants considered for this study had no ocular diseases or systemic diseases known to influence the macula (e.g. diabetic retinopathy, severe hypertension, blood diseases, or cancer). Subclinical cases with discrete maculopathies (e.g. eyes with few small drusen) were not eligible. Sample size calculation was not possible for this hypothesis-generating study due to lack of previous studies on photoreceptors and RPE-BM, aging, and lifestyle. We included 150 participants as our impression is that smaller studies (< 100 participants) are insufficiently powered for aging studies of the macula [10].

### Lifestyle factors

Participants were subjected to a structured interview on four important lifestyle factors: alcohol consumption, physical activity, body mass index (BMI), and smoking habits. Alcohol consumption was reported in units of alcohol per week (1 Danish unit = 15 mL or 12 g of ethanol), which is a commonly used lay term in Denmark. Body mass index (BMI) was calculated using height and weight measurements. Physical activity was evaluated using a yes/no survey question for epidemiological studies as proposed by Schechtman et al. [11]: *Do you currently participate in any regular activity or program (either on your own or in a formal class) designed to improve or maintain your physical fitness?* We translated this question using forward and backward translation [10], and have previously validated its use in elderly Danes [9,12]. Smoking habits were categorized into ever or never smokers, as this was considered a more robust measure towards both recall bias (i.e. inability to accurately report lifetime tobacco use) and reporting difficulties from the relatively frequent use of pipe tobacco and snus (a moist variant of snuff popular in Scandinavian countries) in elderly Danes.

### Imaging protocol

Imaging was made using the Spectralis Spectral-Domain HRA-OCT (Heidelberg Engineering, Heidelberg, Germany). To avoid statistical problems with independent sampling, we selected only one eye for data collection: the eye with the best image quality, and if of equal quality then the right eye [13]. Images were examined in Heidelberg Eye Explorer 1.9.10.0 (Heidelberg Engineering, Heidelberg, Germany). We measured the thickness of the photoreceptors and the RPE-BM at the foveal minimum using the thickness profile part of the software. We also measured the thickness of the photoreceptors and the RPE-BM as a mean of the retinal areas using the thickness map with the following standard definitions: the central subfield (circular area defined by 500 μm radius from the foveal minimum), the inner macula (ring-shaped area that is defined by 500 μm to 1,500 μm radius from the foveal minimum), and the outer macula (ring-shaped area that is defined by 1,500 μm to 3,000 μm radius from the foveal minimum). We used the automatic segmentation feature of the software to distinguish retinal layers. All segmentations were manually checked and corrected where necessary. The software defines the RPE-BM layer from the inner limit of the RPE line to the outer limit of Bruch’s membrane, and the photoreceptor layer from the inner limit of the external limiting membrane to the outer limit of Bruch’s membrane. The latter means that the RPE-BM layer is included in the photoreceptor layer **(Supplementary material 1)**. To differentiate between specific changes in the photoreceptors and the RPE-BM, we calculated photoreceptor-only thickness values for all analyses by subtracting the RPE-BM thickness values from the originally estimated photoreceptor layer values. Since none of our participants had retinal diseases, Bruch’s membrane was not distinguishable from the RPE layer.

### Data analysis and statistics

Normally distributed variables are reported using mean and standard deviation (SD), otherwise using median and interquartile range (IQR). Relationships between the thickness of the photoreceptor layer or the RPE layer with age, sex, and lifestyle factors were investigated using linear regression models. When studying aging, we adjusted values for gender by including it as an independent co-variate since studies suggest gender differences [14]. When studying influence of lifestyle factors, we adjusted values for both age and gender. Alcohol intake had a right-skewed distribution, and the variable was therefore categorized into ≤ 7 units/week (i.e. on average ≤ 1 unit/day) and > 7 units/week (i.e. on average > 1 unit/day) for statistical models. Data were analyzed using SPSS 23 (IBM, Chicago, IL, USA).

## Results

**Figure 1.**
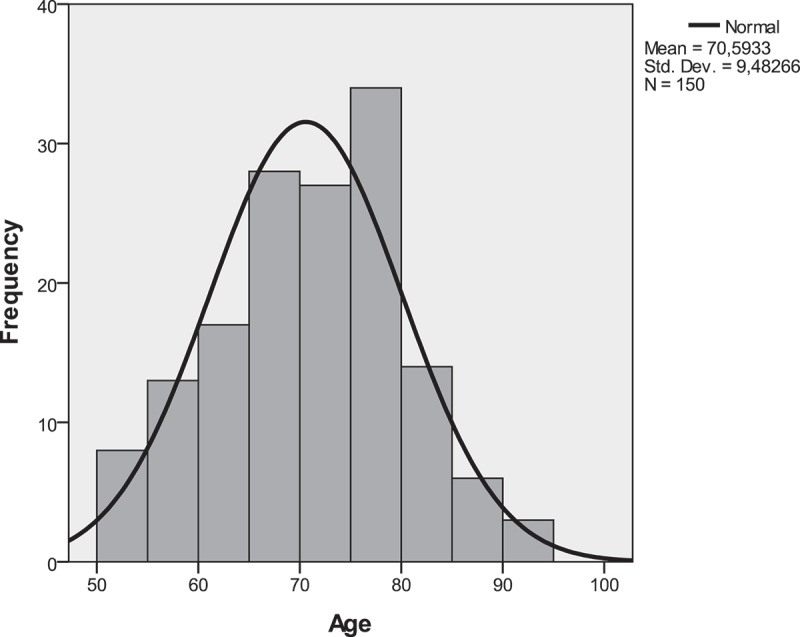
Histogram of the age distribution of participants in this study.

We studied 150 eyes from 150 participants aged mean 70.6 (SD: 9.5) years (range: 50–92 years). Age distribution followed a normal curve (Figure 1): 21 were 50–59 years old, 45 were 60–69 years old, 61 were 70–79 years old, 20 were 80–89 years old, and 3 were 90+ years old. Participant characteristics are summarized in Table 1. Due to the natural curved shape of the normal macula, the foveal minimum (center-point foveal thickness) was significantly thinner than the central subfield thickness (P < 0.001), the central subfield thickness was significantly thinner than the inner (P < 0.001) and outer (P < 0.001) macula, and the inner macula was significantly thicker than the outer macula (P < 0.001).

**Table 1.** Participant characteristics.

Characteristics (150 eyes of 150 participants)	
Demographics	
Age, mean ± SD, years	70.6 ± 9.5
Gender, n (%)	
Females	92 (61)
Males	58 (39)
Lifestyle factors	
Alcohol intake, median (IQR), units/week	5 (1 to 8)
Body mass index, mean ± SD, kg/m^2^	26.1 ± 4.9
Physically active*, n (%)	73 (49)
Smokers, n (%)	
Ever smokers	67 (45)
Never smokers	83 (55)
Macular thickness	
Center-point foveal thickness, mean ± SD, µm	233 ± 24
Central subfield, mean ± SD, µm	285 ± 25
Inner macula, mean ± SD, µm	333 ± 20
Outer macula, mean ± SD, µm	293 ± 18

*No data on 1 participant. Abbreviations: SD = standard deviation; n = numbers; IQR = interquartile range.

### Aging of the photoreceptors and the RPE

**Figure 2.**
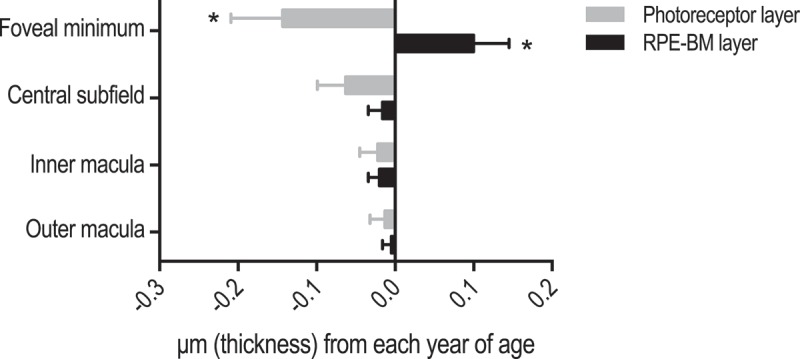
Influence of aging on thickness of the photoreceptor layer and the retinal pigment epithelium/Bruch’s membrane complex (RPE-BM). Bars represent unstandardized regression coefficient and whiskers represent the standard error of the regression coefficient. Estimates are gender-adjusted.

Aging was associated with a decrease of the photoreceptor layer thickness at the foveal minimum (P = 0.031) (Figure 2). This trend was attenuated in the central subfield (P = 0.084), and non-existent in the inner and outer macula. The RPE layer was associated only with an increase with age at the foveal minimum (P = 0.029) (see Figure 2).

### Influence of lifestyle factors

**Figure 3.**
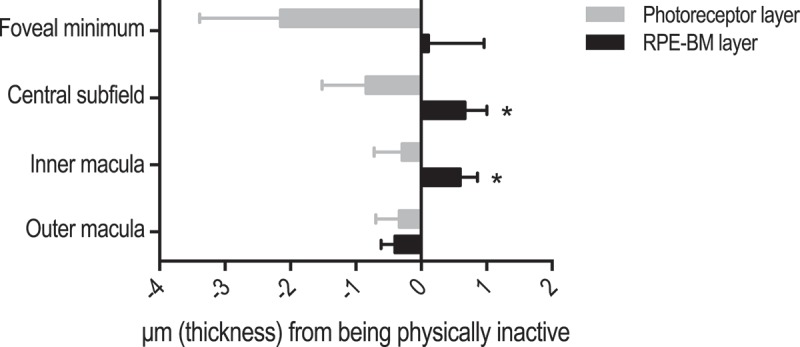
Influence of physical inactivity on thickness of the photoreceptor layer and the retinal pigment epithelium/Bruch’s membrane complex (RPE-BM). Bars represent unstandardized regression coefficient and whiskers represent the standard error of the regression coefficient. Estimates are both age- and gender-adjusted.

**Figure 4.**
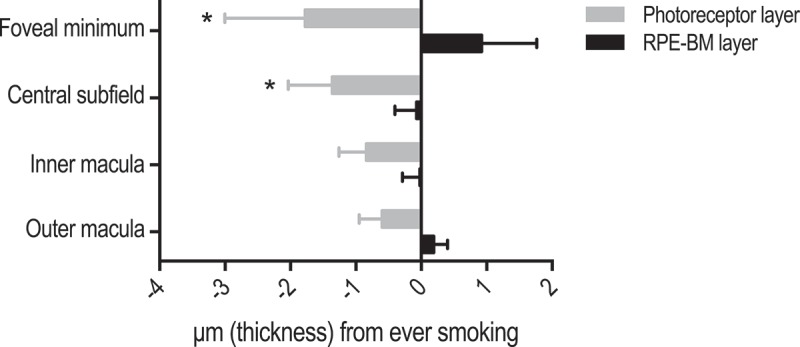
Influence of smoking on thickness of the photoreceptor layer and the retinal pigment epithelium/Bruch’s membrane complex (RPE-BM). Bars represent unstandardized regression coefficient and whiskers represent the standard error of the regression coefficient. Estimates are both age- and gender-adjusted.

Alcohol intake and BMI were not associated with significant differences in the photoreceptor layer or the RPE-BM layer **(Supplementary material 2)**. The physically inactive had a significantly thicker RPE-BM layer in the central subfield (P = 0.044) and in the inner macula (P = 0.025), and a borderline-significant trend towards a thinner photoreceptor layer in the foveal minimum (P = 0.083) (Figure 3). Smokers had significantly thinner photoreceptors in the central subfield (P = 0.044) and in the inner macula (P = 0.048). Other smoking-related trends did not reach statistical significance (Figure 4).

## Discussion

Human maculae are characterized by sharp central vision due to densely packed foveal tissue. In the process of evolution, this has served as a competitive advantage, but at a great price: an extremely high metabolic turnover compared with tissue volume [15] that is managed well in young adults, but not in the aged, since becoming 60+ years old is new in an evolutionary perspective [16–18]. In this *in vivo* study of humans, we found that aging was associated with a thinning of the photoreceptor layer and a thickening of the RPE-BM layer at the foveal minimum. Smoking and physical inactivity were associated with changes that in some respects were similar to that seen with aging.

Histological studies find that, overall, retinal thickness decreases with age [19–21]. By looking at scanning electron photographs of the photoreceptor–RPE junction, Calvalotti et al. found that older donors had fewer synaptic bodies and fewer cellular processes with relatively larger intercellular spaces [19]. The number of RPE cells declines with age, especially in the central part of the macula [19–22]. RPE loss is more pronounced than photoreceptor loss, which leads to an overall increase in the photoreceptor-to-RPE ratio (i.e. number of photoreceptors that each RPE cell must support) [21,22]. RPE cells are terminally differentiated, so when the metabolic stress on the RPE cells increase, the RPE cells do not respond with proliferation but instead respond by increasing the number of nuclei within each cell, which consequently increases the overall cell size [23]. Large multinucleated RPE cells have an increased metabolic demand, which as a consequence increases the reactive oxygen species generated. Exposure to reactive oxygen species may affect the health of photoreceptors and the Bruch’s membrane [24]. RPE isolated from young and old rats share only ~50% of their protein expression and the differences concern mostly proteins involved in pathways of oxidative stress such as catalase, glutathione peroxidase, and superoxidate dismutase [25]. Chen et al. found that exposure to oxidized photoreceptor outer segments induced the multinucleation process in the RPE cells [23], and that age-related RPE multinucleation was more profound in central retina. These findings are in line with our observations in humans *in vivo*. The RPE-BM complex does not seem to increase outside of the foveal minimum and there may even be a decrease with age in the more peripheral areas of the macula [14,26].

Regular physical activity has a well-characterized anti-inflammatory impact on the systemic circulation [27,28]. RPE cells and photoreceptors are sensitive to inflammatory stimulation [29,30], which may explain the differences in thicknesses between the physically active and inactive. Kim et al. investigated these mechanisms in an experimental model, where they evaluated age-related changes on mice allocated either to regular aerobic training (treadmill training) or sedentary controls [31]. The age-related decrease in the overall retinal thickness was smaller in the exercise group [31]. Using immunohistochemistry, the authors also found fewer neuronal cell losses and less oxidative stress (measured using retinal 8-hydroxy-2ʹ-deoxyguanosine) in exercised mice [31].

Smoking has a significant impact on both RPE cells and photoreceptors [32,33]. One example is that smoking leads to abnormal deposits in the RPE cells and disorganizes photoreceptors anatomically [33]. These aspects remain incompletely understood; however, two important functional pathways provide an important theoretical framework. First, smoking mediates a substantial amount of their systemic toxicity though increased systemic reactive oxygen species [34–37]. Second, blood flow studies find decreased retinal blood flow due to increased vascular resistance [38–40], which may exert further pressure on the age-related increased metabolic load on RPE cells.

Relatively fewer studies have looked at Bruch’s membrane in relation to age and lifestyle factors [41]. Histological studies find increased lipid and lipoprotein accumulation with age, which seems to be particularly abundant in individuals aged > 60 years [41]. Using electron microscopy, lipoproteins in aged individuals are seen as spread out in Bruch’s membrane as small particles with a diameter of 60–100 nm [41–43]. In an experimental mouse model, exposure to cigarette smoke thickened Bruch’s membrane due to accumulation of deposits within the membrane [44]. Changes observed in the RPE-BM in our study are more likely to reflect changes in the RPE due to size differences (Bruch’s membrane (2–4 μm) is much thinner than the RPE), which means that changes in the RPE may contribute relatively more; however, the overall direction of changes seems to be similar in both the RPE and Bruch’s membrane.

Two important limitations of this study should be noted. First, this was a cross-sectional study, which can only associate and not infer on causality. Second, alcohol intake, smoking status, and physical activity were all obtained using simple questions which can provide directions, whereas more detailed questionnaires or accelerometers allow for more precise and detailed associations. Such data could give more detailed insights into smoking habits and physical activity, which could enable investigations of a possible dose–effect relationship.

Taken together, our findings suggest that smoking and physical inactivity may partly accelerate an aging process of the photoreceptors and the RPE-BM of the macula that is predominantly a thickness decrease of photoreceptors and a thickness increase of RPE-BM. Aging processes play a significant role on macular health and warrant further investigations. Our findings contribute to the understanding of how lifestyle factors such as smoking and physical activity contribute to promotion of age-related retinal diseases such as age-related macular degeneration [2,3].

## Supplementary Material

supplementary_material.zip
