# Supplementary figures and images for: Effect of aging and lifestyle on photoreceptors and retinal pigment epithelium: cross-sectional study in a healthy Danish population
Source: Pathobiol Aging Age Relat Dis. 2017 Nov 5;7(1):1398016. doi: 10.1080/20010001.2017.1398016 (PMC5678353; doi:10.1080/20010001.2017.1398016)

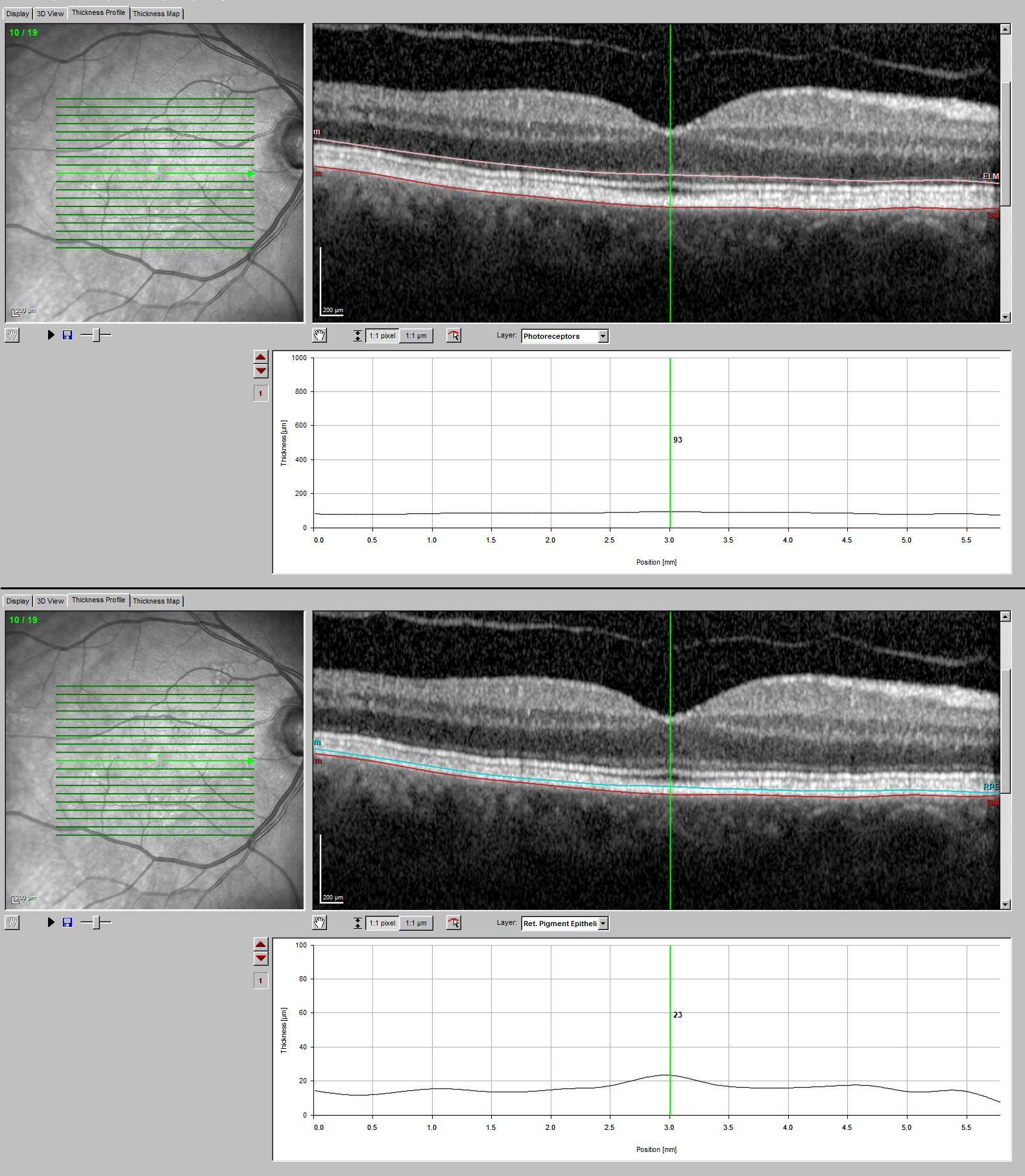

Supplement: supplementary_material.zip [file ZPBA_A_1398016_SM3116.zip › supplementary material/Supplementary material 1.tif]
